# Celecoxib Decreases the Need for Rescue Analgesics after Total Knee Arthroplasty: A Meta-Analysis

**DOI:** 10.3390/clinpract14020035

**Published:** 2024-03-18

**Authors:** Eduardo Gómez-Sánchez, Adriana Hernández-Gómez, Juan Manuel Guzmán-Flores, Angel Josabad Alonso-Castro, Nicolás Addiel Serafín-Higuera, Luz Ma.-Adriana Balderas-Peña, Lorenzo Franco-de la Torre, Mario Alberto Isiordia-Espinoza

**Affiliations:** 1 División de Disciplinas Clínicas, División de Disciplinas Básicas para la Salud, Cuerpo Académico UDG-CA-874 Ciencias Morfológicas en el Diagnóstico y Tratamiento de la Enfermedad, Centro Universitario de Ciencias de la Salud, Universidad de Guadalajara, Guadalajara 44340, Mexico; eduardo.gsanchez@academicos.udg.mx (E.G.-S.); luz.balderas@academicos.udg.mx (L.M.-A.B.-P.); 2 Departamento de Ciencias de la Salud, División de Ciencias Biomédicas, Centro Universitario de los Altos, Universidad de Guadalajara, Tepatitlán de Morelos 47620, Mexico; adriana.hgomez@academicos.udg.mx (A.H.-G.); jmanuel.guzman@academicos.udg.mx (J.M.G.-F.); 3 Instituto de Investigación en Ciencias Médicas, Cuerpo Académico Terapéutica y Biología Molecular (UDG-CA-973), Departamento de Clínicas, División de Ciencias Biomédicas, Centro Universitario de los Altos, Universidad de Guadalajara, Tepatitlán de Morelos 47620, Mexico; lorenzo.franco@academicos.udg.mx; 4 Departamento de Farmacia, División de Ciencias Naturales y Exactas, Universidad de Guanajuato, Guanajuato 36250, Mexico; angeljosabad@ugto.mx; 5 Facultad de Odontología Mexicali, Universidad Autónoma de Baja California, Mexicali 21040, Mexico; nicolas_addiel@yahoo.com.mx; 6 Unidad de Investigación Biomédica 02, UMAE Hospital de Especialidades, Centro Médico Nacional de Occidente, Instituto Mexicano del Seguro Social, Guadalajara 44340, Mexico

**Keywords:** celecoxib, placebo, analgesic efficacy, adverse effects, total knee arthroplasty

## Abstract

This systematic review and meta-analysis aimed to evaluate the analgesic efficacy and adverse effects of celecoxib after total knee arthroplasty. Keywords in the PubMed and Scopus databases were used to find article abstracts. Each included clinical trial was assessed using the Cochrane Collaboration risk of bias tool, and we extracted data on postoperative pain assessment using the Visual Analogue Scale (VAS) at rest, ambulation, and active range of motion, rescue analgesic intake, and adverse effects. Inverse variance tests with mean differences were used to analyze the numerical variables. The Mantel–Haenszel statistical method and the odds ratio were used to evaluate the dichotomous data. According to this qualitative assessment (*n* = 482), two studies presented conclusions in favor of celecoxib (*n* = 187), one showed similar results between celecoxib and the placebo (*n* = 44), and three clinical trials did not draw conclusions as to the effectiveness of celecoxib versus the placebo (*n* = 251). Moreover, the evaluation of the rescue analgesic intake showed that the patients receiving celecoxib had a lower intake compared to patients receiving a placebo (*n* = 278, I^2^ = 82%, *p* = 0.006, mean difference = −6.89, 95% IC = −11.76 to −2.02). In conclusion, the pooled analysis shows that administration of celecoxib alone results in a decrease in rescue analgesic consumption compared to a placebo after total knee surgery.

## 1. Introduction

Acute pain after total knee arthroplasty is very intense and disabling for patients undergoing this surgical procedure [1,2,3]. This can hinder the patient’s mobility in terms of both passive and active movement, support while walking or resting, and stiffness in the joints. This lack of mobility, consequently, delays the patient’s recovery, affecting the quality of life [4,5,6,7,8].

Advances in clinical, surgical, and pharmacological procedures have allowed better pain management in patients undergoing total knee arthroplasty [2,8,9,10]. Recommendations include the use of a multimodal approach using different types of pain management medications, such as opioid analgesics, nonsteroidal anti-inflammatory drugs (NSAIDs), glucocorticosteroids, gabapentinoids, and anesthetics—i.e., bupivacaine hydrochloride—[8,9,10,11]. On the other hand, the use of pharmacological monotherapy for pain management after this type of surgical procedure is questionable [12].

The use of COX-2 selective NSAIDs before, during, and after total knee arthroplasty is a relatively common choice that would imply advantages due to the nature of the type of pain suffered by the patient [13]. However, high concentrations of this drug also produce inhibition of the COX-1 enzyme using in vitro assays. Preclinical studies have shown that the therapeutic plasma concentration of celecoxib should be approximately 300 ng/mL, and single-dose pharmacokinetic studies in humans have suggested that doses as low as 100 mg of celecoxib would achieve this concentration [14]. Currently, there is no systematic review with meta-analysis that evaluates the individual effect of celecoxib in total knee arthroplasty; so, this study aims to compile the best scientific evidence available to provide the clinician with a real view of the analgesic efficacy and adverse effects of this drug after total knee arthroplasty.

## 2. Materials and Methods

### 2.1. Population, Interventions, Control, and Outcome Strategy [15]

#### 2.1.1. Inclusion Criteria

Population: clinical trials included patients undergoing total knee arthroplasty;
Interventions: patients received celecoxib;
Control: patients received a placebo;
Outcome: evaluation of postoperative pain using the Visual Analogue Scale (VAS) at rest, ambulation, and active range of motion, rescue analgesic intake, and adverse effects.

#### 2.1.2. Exclusion Criteria

RCT with a loss to follow-up greater than 20%.

### 2.2. Research Question

What are the analgesic and adverse effects of celecoxib and placebo after total knee arthroplasty?

### 2.3. Information Search

Studies published from 2000 to July 2023 were considered. The following terms were used in the PubMed and Scopus databases to find abstracts of clinical trials related to the keywords: “celecoxib” AND “total knee arthroplasty”; “celecoxib” AND “orthopedic surgery”; “COX-2 inhibitor” AND “total knee arthroplasty”; “COX-2 inhibitor” AND “orthopedic surgery”; “NSAIDs” AND “total knee arthroplasty”; and “NSAIDs” AND “orthopedic surgery”. PubMed filters for study type/design and language (“English” and “Spanish”) were used. The running protocol was sent to and accepted by the PROSPERO system from the University of York (ID CRD42023486909).

### 2.4. Assessment of Bias

Each clinical trial was assessed using the Cochrane Collaboration risk of bias tool [16,17,18,19]. The evaluations were carried out by two independent evaluators [16,17,18,19]. The decision on the qualification of each evaluating study was made by consensus between both participants, and when there was a difference between them, a third evaluator participated to reach a majority decision [16,17,18,19].

### 2.5. Data Extraction

The data were recorded in an Excel database and subsequently moved to a statistical program. The data included the author, study design, treatment groups, sample size (*n*), dose, evaluation of postoperative pain with the VAS in rest, ambulation, and active range of motion, rescue analgesic intake, and adverse effects.

### 2.6. Statistical Analysis

The inverse variance test with means difference was employed to analyze the numerical variables. The Mantel–Haenszel statistical method and odds ratio (OR) were used to evaluate the dichotomous data. Moreover, the heterogeneity was evaluated as previously reported in a published article [20]. All meta-analyses were conducted using a random effect model with the Review Manager Software 5.3 for Windows. A *p* value overall statistical test <0.05 and an OR > 1 with 95% confidence intervals (CI) in each meta-analysis were considered as statistical differences [16,21,22].

Sensitivity analysis was performed to observe variations in the statistical analysis when statistical differences were obtained in the meta-analyses and to understand the influence that each study had on the results of the pooled data [23].

## 3. Results

### 3.1. Information Search

**Figure 1:**
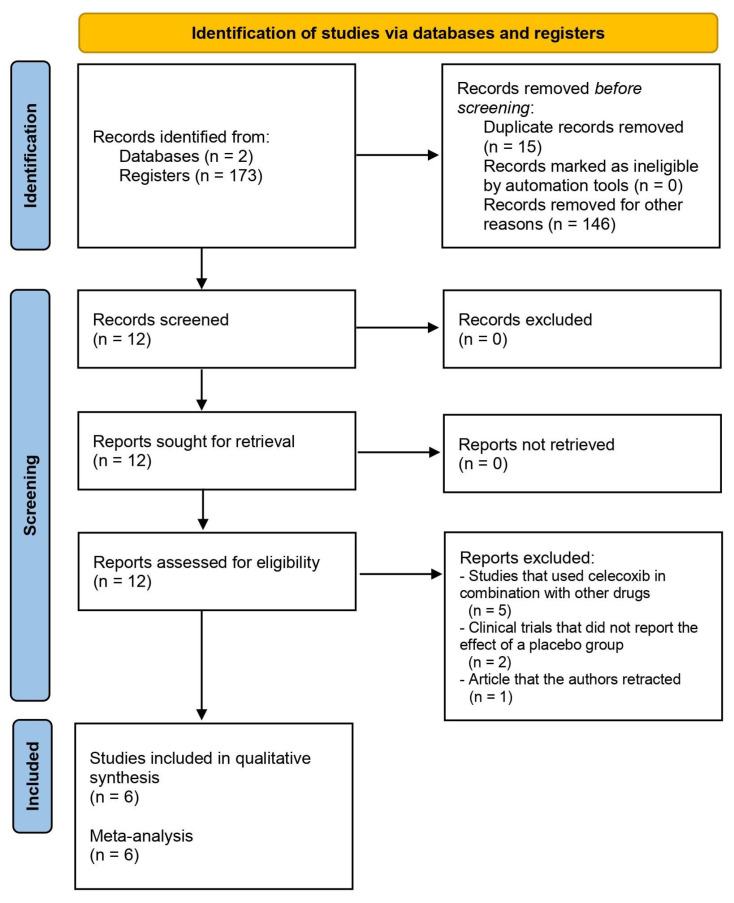
Study flow diagram.

The search in the databases used in this systematic review resulted in 173 articles related to the different groups of keywords used. Fifteen duplicate reports were removed, and 146 articles were removed for other reasons. As Figure 1 and Table 1 show, six clinical trials [24,25,26,27,28,29] were included in the qualitative analysis of this systematic review.

### 3.2. Bias Assessment

**Figure 2:**
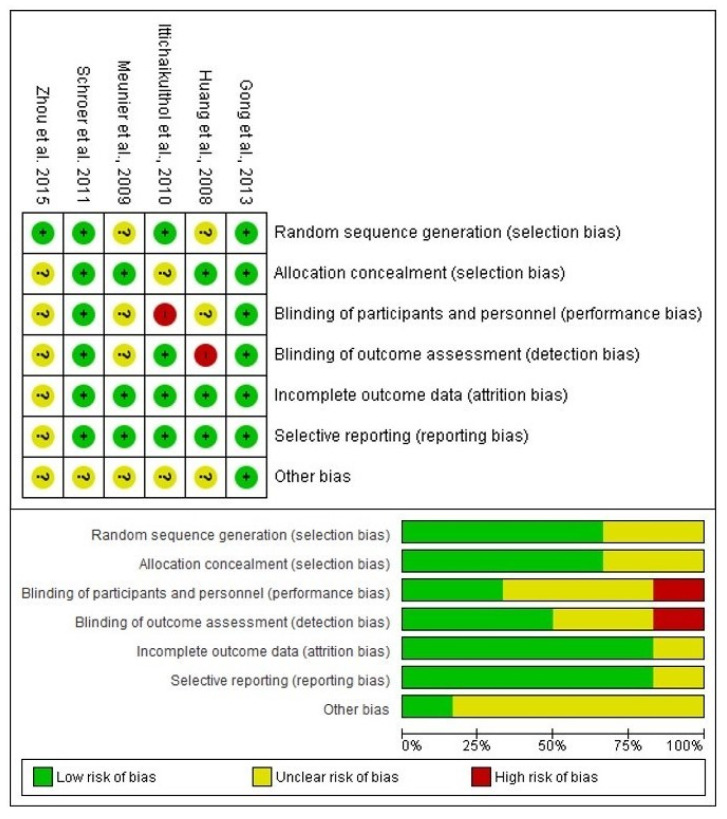
Assessment of risk of bias [24,25,26,27,28,29].

The risk of bias assessment included a total of six scientific reports evaluating the efficacy of celecoxib compared to a placebo in total knee arthroplasty. The results of the risk of bias assessment showed that four articles [24,27,28,29] had a low to moderate risk because they did not obtain red circles in their evaluations. However, two of those clinical trials had a high risk of bias [25,26]. The reason for this high risk of bias was the lack of blinding of participants, staff, and the clinical evaluator who collected the data (Figure 2).

### 3.3. Qualitative Assessment

The qualitative evaluation of the studies was carried out considering the conclusion of each of the articles (*n* = 482) [24,25,26,27,28,29]. According to this section, two studies presented conclusions in favor of celecoxib (*n* = 187) [25], one showed similar results between celecoxib and the placebo (*n* = 44) [27], and three clinical trials did not conclude as to the effectiveness of celecoxib versus the placebo (*n* = 251) [24,26,29] because they had another objective, i.e., two studies had as their main objective to compare the effectiveness of drug combinations in total knee arthroplasty, and one concluded that another analgesic was superior to celecoxib and the placebo.

### 3.4. Quantitative Evaluation

**Figure 3:**
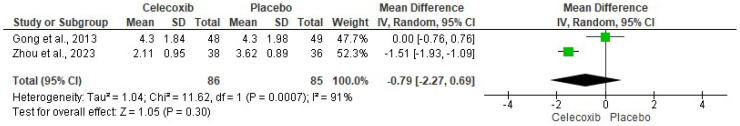
Evaluation of postoperative pain with the limb at rest [24,29].

**Figure 4:**
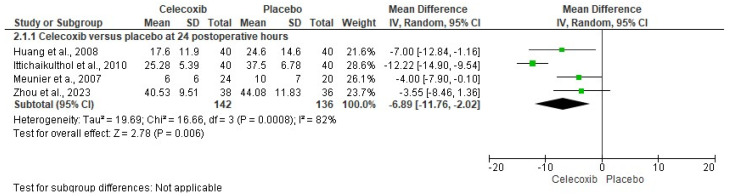
Analysis of pooled data on rescue analgesic intake in patients receiving celecoxib versus a placebo after total knee arthroplasty [25,26,27,29].

**Figure 5:**
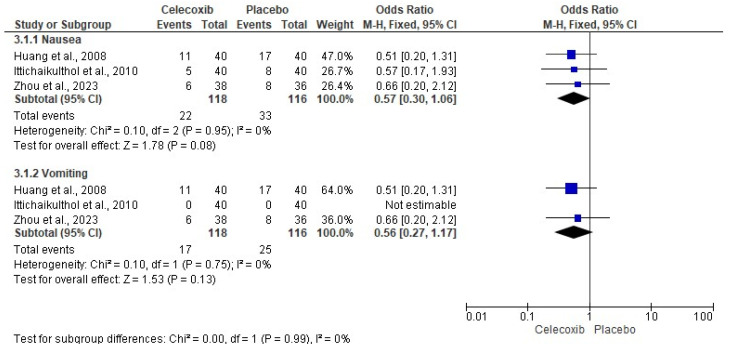
Meta-analysis of the adverse effects of celecoxib [25,26,29].

The assessment of pain intensity with the VAS at rest was performed using data from two clinical trials [24,29]. Analysis of the data showed no statistical differences between celecoxib and the placebo (*n* = 171, I^2^ = 91%, *p* = 0.3, mean difference = −0.79, 95% IC = −2.27 to 0.69; Figure 3). On the other hand, the evaluation of the intake of rescue analgesics was carried out with four clinical trials [25,26,27,29]. The statistical evaluation showed that the patients receiving celecoxib had a lower rescue analgesic intake compared to patients receiving a placebo (*n* = 278, I^2^ = 82%, *p* = 0.006, mean difference = −6.89, 95% IC = −11.76 to −2.02; Figure 4). Finally, the adverse reactions were evaluated using information from three clinical trials (*n* = 234) [25,26,29]. Both nausea (*n* = 234, I^2^ = 0%, *p* = 0.08, OR = 0.57, 95% IC = 0.3 to 1.06; Figure 5) and vomiting (*n* = 234, I^2^ = 0%, *p* = 0.13, OR = 0.56, 95% IC = 0.27 to 1.17; Figure 5) were similar between celecoxib and a placebo.

### 3.5. The Sensitivity Assessment and Publication Bias

**Figure 6:**
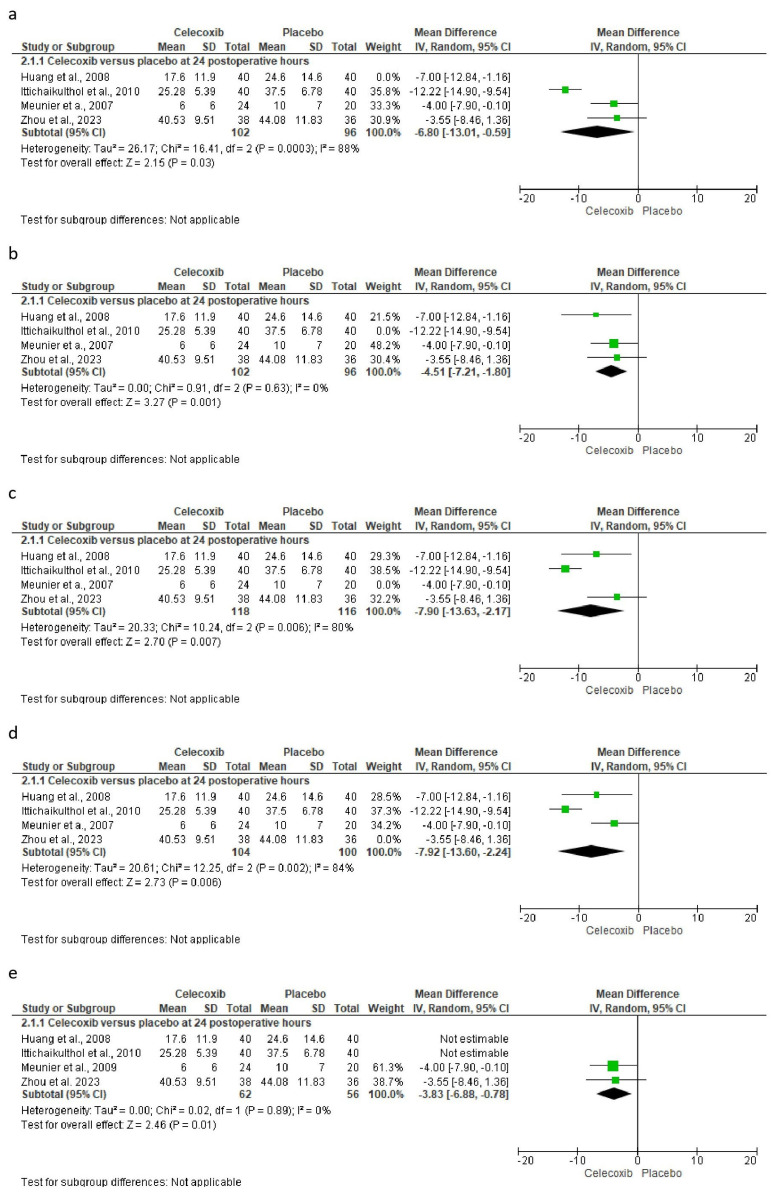
Sensitivity analysis shows how the statistical difference is preserved even when data from different studies are excluded. Excluded study: (**a**) = Huang et al., 2008; (**b**) = Ittichaikulthol et al., 2010; (**c**) = Meunier et al., 2009; (**d**) = Zhou et al., 2023; and (**e**) = Huang et al., 2008 and Ittichaikulthol et al., 2010 [25,26,27,29].

The sensitivity analysis was carried out only for the consumption of rescue analgesics, which did not show variability in the results. That is, despite having performed this sensitivity analysis, the results maintained the statistical difference (Figure 6) [25,26,27,29].

## 4. Discussion

This is the first systematic review and meta-analysis that evaluates the analgesic efficacy as well as the safety of only celecoxib following total knee surgery. The most important result of this systematic review is the decreased consumption of postoperative analgesics in patients who received celecoxib compared to those who received a placebo. It is important to highlight that this same variable was used to carry out the sensitivity analysis in which it was observed that despite extracting the different trials to carry out the statistical analysis and even having excluded from the analysis those studies with a high risk of bias, the statistical difference was maintained during all executions.

During the full reading of the articles, several clinical trials were excluded for different reasons: studies that used celecoxib in combination with other drugs [30,31,32,33,34], clinical trials that did not report the effect of a placebo group [35,36], and an article that the authors retracted [37]. That is, they did not report the therapeutic effect of celecoxib alone after total knee arthroplasty. Moreover, the assessment of the risks of bias in the clinical trials included in this systematic review and meta-analysis showed a moderate risk of bias in four studies [24,27,28,29], and two clinical trials had a high risk of bias [25,26]. Blinding of participants and personnel, as well as blinding of outcome assessment, were the reasons why these last two clinical trials were considered at high risk of bias.

The qualitative evaluation of the results showed that celecoxib produced a better analgesic effect compared with a placebo after total knee surgery. Two studies showed favorable results for celecoxib [25,28], one reported similar analgesic efficacy to a placebo [27], and in the remaining three, although they did not conclude in terms of the use of celecoxib, the detailed analysis of the information clearly showed that celecoxib was better than a placebo after total knee surgery [24,26,29].

The quantitative evaluation showed that celecoxib presented a statistically significant decrease in postoperative rescue analgesic consumption compared to a placebo after this type of surgery. It is important to note that the heterogeneity of this meta-analysis was high, and to be conservative in our statistical analysis, the random effects model was used. The decreased consumption of rescue analgesics in the postoperative period is an important finding that has been observed in many surgical areas [38,39,40,41,42,43,44,45].

Jiang et al., 2020 performed a systematic review and meta-analysis on the analgesic efficacy and adverse effects of COX-2 enzyme inhibitors in total knee and hip arthroplasty [46]. The authors observed a statistical difference in several indicators of clinical effectiveness, such as pain perception at rest and while walking, a decrease in postoperative opioid analgesic consumption, and the incidence of nausea and fever. When comparing our systematic review with that conducted by Jiang et al., 2020 [46], we observed some important differences. The first difference is that our study reports the analgesic efficacy and adverse effects of celecoxib alone compared with a placebo, and the study by Jiang et al., 2020 [46] showed the overall effect of COX-2 enzyme inhibitors after total knee surgery. In our study, we only observed that a single variable obtained a statistical difference; we observed a decrease in the consumption of rescue analgesics in the postoperative period. Jiang et al., 2020 [46] found differences in five variables for which it was possible to combine the data to perform a statistical analysis. Furthermore, the sample size of our study was smaller than that of the aforementioned study [46]. Hong et al. performed a systematic review and meta-analysis to determine the analgesic efficacy and adverse effects of parecoxib compared with a placebo. The authors reported that parecoxib produced better pain relief 24 h postoperatively compared with a placebo, while adverse effects such as nausea and vomiting were similar between both groups [47]. Moreover, Geng et al., 2022 performed a systematic review and meta-analysis on the use of celecoxib in total knee arthroplasty, and their results showed statistical differences in the intensity of extremity pain at rest, a decrease in the consumption of opioid analgesics, and a greater range of active motion. However, several of the studies included in their statistical analysis used a combination of celecoxib with another drug [30,33,34]; that is, the analgesic effect and adverse effects of celecoxib alone in total knee arthroplasty were not evaluated [48].

NSAIDs selective for the COX-2 enzyme reduce the risk of gastropathies, as well as kidney and cardiovascular damage—cerebrovascular or myocardial infarction—because they do not inhibit COX-1 [49,50,51,52,53,54]. Selective inhibition of COX-2 produces an analgesic and anti-inflammatory effect, because this COX-2 enzyme produces prostaglandins and other byproducts of arachidonic acid [49,50,51,52,53].

Pain is a subjective phenomenon that varies greatly between each subject. Making a comparison between a pharmacological treatment and a placebo is key for many studies that evaluate analgesia since it offers many advantages, from methodological to statistical, in randomized clinical trials. In this particular case, the comparison with a placebo gives us the possibility to calculate the NNT and CI for this particular drug, which would have allowed valid indirect comparisons with other analgesic treatments using the NNT and CI [54,55,56,57,58,59,60]. Unfortunately, none of the variables including the number of patients presented statistical differences in the meta-analysis; so, it was not possible to calculate these analgesic efficacy estimators that would have been of great interest to physicians.

The main advantage of this study is that it reports the analgesic efficacy and adverse effects of celecoxib alone compared to a placebo. The strengths of this systematic review are the statistics, which were performed conservatively, always considering the result of heterogeneity, as well as the sensitivity analysis [16,20,21,22,58,59]. The main disadvantage of this study is the limited number of studies that met the selection criteria, which allowed for a relatively small sample size, as well as the design of this type of study—retrospective—[60]. Another limitation of this systematic review was the language since only studies in Spanish and English were included. The study by Jiang et al. included some Chinese language studies that were not found by our electronic search team and that would have allowed a larger sample size [46]. In addition, another important limitation of this study was the calculation of clinical effectiveness estimators, such as the number needed to treat and confidence intervals, which could not be calculated because the data were not presented as frequencies.

In conclusion, we can highlight that there is evidence of moderate quality from the pooled analysis of data from the studies included in this systematic review and meta-analysis that shows that administration of celecoxib alone results in a decrease in rescue analgesic consumption compared to a placebo after total knee surgery.

## Figures and Tables

**Table 1:** Details of the included clinical trials.

ID Study and Study Design	Treatments (*n*)	Details of Patients, Surgical Procedure, and Evaluation	Authors’ Conclusion
Gong et al., 2013 [24]. Randomized, double-blind, placebo-controlled, and parallel-group clinical trial.	Group A: Celecoxib 300 mg twice daily orally and eperisone hydrochloride 50 mg thrice daily orally for 14 days (*n* = 50). Group B: Celecoxib 300 mg twice daily orally and placebo thrice daily (*n* = 48). Group C: Placebo (*n* = 49).	Patients with degenerative arthritis in the knee joint with grade III muscle strength and between 50 and 75 years of age who required total knee arthroplasty were included. The surgery was performed under general anesthesia, was controlled with a tourniquet, and used in all cases a CR prosthesis (Gemini, MK-II. Link). A thromboprophylaxis protocol of rivaroxaban 10 mg per day was used. Morphine (patient-controlled analgesia) was used during the hospital stay. Antibiotic prophylaxis with vancomycin or cefotaxime was used. Pain intensity at rest (VAS score), ambulation (VAS score), active range of motion, postoperative morphine intake, and adverse effects were evaluated.	The celecoxib–eperisone combination had better analgesic efficacy compared to celecoxib alone and the placebo.
Huang et al., 2008 [25]. Randomized, observer-blind, and parallel-group clinical study.	Group A: Single 400 mg dose of celecoxib 1 h before surgery and 200 mg of celecoxib every 12 h for five days, along with patient-controlled analgesic morphine (*n* = 40). Group B: Patient-controlled analgesic morphine alone (*n* = 40).	Patients with osteoarthritis and those over sixty years of age were included. Spinal anesthesia was used. No description of the surgical procedure was provided. Morphine rescue analgesia was utilized. The VAS pain scores, active range of motion, total opioid intake, and adverse effects were evaluated.	Celecoxib was better in all efficacy indicators compared to the control group.
Ittichaikulthol et al., 2010 [26]. Randomized, single-blind, placebo-controlled, and parallel-group clinical assay.	Group A: Celecoxib 400 mg orally (*n* = 40). Group B: Parecoxib 40 mg IV (*n* = 40). Group C: Placebo orally (*n* = 40).	ASA-1 and -2 patients, aged 18 to 75 years, for elective knee surgery were included. For general anesthesia, intravenous thiopental 3–5 mg/kg and fentanyl 2 mcg/kg were used and maintained with sevoflurane and 66% nitrous oxide in oxygen. No description of the surgical procedure was provided. Morphine rescue analgesia was employed. Pain scores, morphine intake, sedation scores, and adverse effects were evaluated.	Parecoxib had a better analgesic effect than celecoxib and the placebo.
Meunier et al., 2007 [27]. Randomized, double-blind, placebo-controlled, and parallel-group clinical trial.	Group A: Celecoxib 200 mg (*n* = 24). Group B: Placebo (*n* = 20). Both treatments were given orally 1 h preoperatively and then twice daily for 3 weeks.	Patients from age 50 to 80 years, ASA-1 and -2, and capacity to give informed consent. Prophylactic treatment with 2 g intravenous cloxacillin was used. Subarachnoid spinal anesthesia with isobaric bupivacaine was used. Sedation with midazolam was used only if necessary. The procedures were performed by three surgeons. Pain VAS scores, active range of motion, and ketobemidone intake were assessed.	Similar clinical efficacy was observed between celecoxib and the placebo.
Schroer et al., 2011 [28]. Randomized, double-blind, placebo-controlled, and parallel-group clinical assay.	Group A: Celecoxib 200 mg orally twice daily for six weeks (*n* = 53). Group B: Placebo orally (*n* = 54)	Spinal anesthesia was used. The surgical procedures were conducted by the same surgeon with a minisubvastus technique. Morphine (patient-controlled analgesia) was used during hospital stay. Narcotic intake, knee flexion, Knee Society Score, Oxford Knee Score, and Short-Form 12 scores were evaluated.	Celecoxib induced better postoperative pain relief and faster recovery than the placebo.
Zhou et al., 2023 [29]. Randomized, double-blind, placebo-controlled, and parallel-group clinical study.	Group A: Celecoxib 200 mg and placebo 150 mg (*n* = 38). Group B: Pregabalin 150 mg and placebo 200 mg (*n* = 38). Group C: Celecoxib 200 mg and pregabalin 150 mg (*n* = 37). Group D: Placebo 200 mg and placebo 150 mg (*n* = 36). The treatments were given orally 2 h before surgery.	Patients who received elective, initial, and single total knee arthroplasty treatment were included. ASA-4 patients were excluded. General anesthesia before surgery was used, and a peripheral infiltration (0.5% ropivacaine, 10 mg/mL triamcinolone acetonide acetate, 0.1% epinephrine hydrochloride) was given to all patients after surgery. No description of the surgical procedure was provided. Pain scores at rest and with movement, opioid intake, hs-CRP level, maximal knee flexion range of motion, time to first analgesia after surgery, and adverse effects were evaluated.	The drug combination had a better analgesic effect and decreased the intake of analgesics after surgery.

VAS = Visual Analogue Scale, CR = cruciate-retained, ASA = American Society of Anesthesiologists, CRP = C-reactive protein.

## Data Availability

The data included in this study were extracted from articles that met the selection criteria of our study. Data can be found available in these clinical trials and in the files generated to perform meta-analyses.
